# Pharmacokinetics of ivermectin metabolites and their activity against *Anopheles stephensi* mosquitoes

**DOI:** 10.1186/s12936-023-04624-0

**Published:** 2023-06-24

**Authors:** Charlotte Kern, Pie Müller, Carlos Chaccour, Matthias E. Liechti, Felix Hammann, Urs Duthaler

**Affiliations:** 1 Division of Clinical Pharmacology & Toxicology, Department of Internal Medicine, University Hospital Bern, Bern, Switzerland; 2 Graduate School for Health Sciences, University of Bern, Bern, Switzerland; 3 Swiss Tropical and Public Health Institute, Allschwil, Switzerland; 4 University of Basel, Basel, Switzerland; 5 ISGlobal, Hospital Clínic - Universitat de Barcelona, Barcelona, Spain; 6 Centro de Investigación Biomédica en Red de Enfermedades Infecciosas, Madrid, Spain; 7 Facultad de Medicina, Universidad de Navarra, Pamplona, Spain; 8 Division of Clinical Pharmacology & Toxicology, Department of Biomedicine, University and University Hospital Basel, Basel, Switzerland; 9 Division of Clinical Pharmacology & Toxicology, Department of Pharmaceutical Sciences, University of Basel, Basel, Switzerland

## Abstract

**Background:**

Ivermectin (22,23-dihydroavermectin B_1a_: H_2_B_1a_) is an endectocide used to treat worm infections and ectoparasites including lice and scabies mites. Furthermore, survival of malaria transmitting *Anopheles* mosquitoes is strongly decreased after feeding on humans recently treated with ivermectin. Currently, mass drug administration of ivermectin is under investigation as a potential novel malaria vector control tool to reduce *Plasmodium* transmission by mosquitoes. A “post-ivermectin effect” has also been reported, in which the survival of mosquitoes remains reduced even after ivermectin is no longer detectable in blood meals. In the present study, existing material from human clinical trials was analysed to understand the pharmacokinetics of ivermectin metabolites and feeding experiments were performed in *Anopheles stephensi* mosquitoes to assess whether ivermectin metabolites contribute to the mosquitocidal action of ivermectin and whether they may be responsible for the post-ivermectin effect.

**Methods:**

Ivermectin was incubated in the presence of recombinant human cytochrome P_450_ 3A4/5 (CYP 3A4/5) to produce ivermectin metabolites. In total, nine metabolites were purified by semi-preparative high-pressure liquid chromatography. The pharmacokinetics of the metabolites were assessed over three days in twelve healthy volunteers who received a single oral dose of 12 mg ivermectin. Blank whole blood was spiked with the isolated metabolites at levels matching the maximal blood concentration (C_max_) observed in pharmacokinetics study samples. These samples were fed to *An. stephensi* mosquitoes, and their survival and vitality was recorded daily over 3 days.

**Results:**

Human CYP3A4 metabolised ivermectin more rapidly than CYP3A5. Ivermectin metabolites M1–M8 were predominantly formed by CYP3A4, whereas metabolite M9 (hydroxy-H_2_B_1a_) was mainly produced by CYP3A5. Both desmethyl-H_2_B_1a_ (M1) and hydroxy-H_2_B_1a_ (M2) killed all mosquitoes within three days post-feeding, while administration of desmethyl, hydroxy-H_2_B_1a_ (M4) reduced survival to 35% over an observation period of 3 days. Ivermectin metabolites that underwent deglycosylation or hydroxylation at spiroketal moiety were not active against *An. stephensi* at C_max_ levels. Interestingly, half-lives of M1 (54.2 ± 4.7 h) and M4 (57.5 ± 13.2 h) were considerably longer than that of the parent compound ivermectin (38.9 ± 20.8 h).

**Conclusion:**

In conclusion, the ivermectin metabolites M1 and M2 contribute to the activity of ivermectin against *An. stephensi* mosquitoes and could be responsible for the “post-ivermectin effect”.

**Supplementary Information:**

The online version contains supplementary material available at 10.1186/s12936-023-04624-0.

## Background

*Plasmodium* parasites are the causative agents of malaria, and are transmitted to humans through the bites of female *Anopheles* mosquitoes. Although the disease is both preventable and curable, malaria remains a significant global public health problem. Globally, there were an estimated 241 million malaria cases in 2020, 6% more than in 2019, and deaths increased by 12% to about 627,000 deaths, mainly due to service disruptions during the COVID-19 pandemic [1, 2]. At the beginning of the twenty-first century, the large-scale implementation of preventive strategies such as long-lasting insecticidal nets and indoor residual spraying led to a decline in malaria incidence. However, the emergence of resistance to insecticides and treatments in mosquitoes and parasites, respectively, is problematic and hampers malaria vector control interventions. Therefore, additional approaches are needed to reduce the global malaria burden. Following the World Health Organization Global Technical Strategy for Malaria 2015–2030, a roadmap was drafted by global health experts for the development of ivermectin as a potential complementary malaria vector control tool [3].

Ivermectin is a broad-spectrum antiparasitic drug, used in a wide range of infestations such as helminths, scabies, and mites. It has been licensed for human use for more than thirty years. Its safety profile has been thoroughly assessed in over 70 trials and extensive post-marketing surveillance. Billions of doses have been distributed worldwide for the control of neglected tropical diseases with a good safety profile, with the important exception of its use in *Loa loa* co-endemic regions [3–8].

After a mosquito has ingested ivermectin with a blood meal from a treated subject, ivermectin binds to the glutamate-gated chloride ion channels, leading to hyperpolarisation of the neuronal membrane, paralysing and potentially even killing the insect [9]. Numerous studies have reported the lethal effect of ivermectin in different mosquito species, such as *Anopheles gambiae, Anopheles albimanus*, *Anopheles arabiensis* and *Anopheles stephensi* [3, 4, 10–15]. Exposure to ivermectin concentrations in the low ng/mL range decreases the survival and fertility of mosquitoes. The concentration that kills 50% of mosquitoes (LC_50_) within 7-days ranges from 3 to 55 ng/mL in *Anopheles* spp., and 178–187 ng/mL in *Aedes aegypti* [16–19].

Approximately 4 h after a single oral dose of 150 µg/kg ivermectin, the peak concentration is around 40 ng/mL [20, 21]. After giving a single oral dose of ivermectin to humans, the drug levels in human capillary blood, from where mosquitoes feed, can be maintained well above LC_50_ for a considerable time [4, 11, 22–24]. In addition, the mean elimination half-life of ivermectin ranges from 25 to 80 h, and effective concentrations are thus maintained for several days post-administration [21, 25]. Ivermectin, with its sound safety profile in humans, unique mode of delivery, novel mode of action, and long plasma half-life, is a promising candidate as a first-in-class malaria vector control tool. Currently, several clinical trials are investigating the effect of ivermectin mass drug administration on malaria transmission by diminishing the mosquito population in endemic regions [3, 26–29]. The community delivery of ivermectin could address residual transmission, which is defined as a sustained transmission even after reaching an appropriate coverage with standard vector control tools. Residual transmission is mostly driven by mosquitoes biting outdoors, early in the evening or both, and feeding on livestock as an ecological niche [30, 31]. A community delivery to both humans and livestock would likely increase MDA efficacy, as mosquitoes feeding on treated animals would also be exposed to ivermectin. This is considered in the design of current MDA campaigns trials such as BOHEMIA [32].

*Anopheles stephensi* is expanding its geographic range worldwide and has been implicated in outbreaks of urban malaria [33]. The World Health Organization (WHO) published a vector alert calling for active mosquito surveillance in Ethiopia and Sudan after an unusual outbreak of urban malaria. In December 2022, *An. stephensi* was detected in Kenya for the first time [34].

Interestingly, the blood of treated individuals reduces the survival of the mosquitoes beyond what is expected from plasma pharmacokinetics of the parent compound, even up to 28 days post-dosing [11], which is longer than expected considering the estimated half-life of ivermectin (1–3 days) [25]. As ivermectin is highly lipophilic and protein-bound (> 90%) [35, 36], its pharmacokinetic patterns can differ according to several factors, such as sex, body mass index and feeding state, with a higher body fat percentage providing larger peripheral volume of distribution in female subjects, and concurrent food intake affecting gastro-intestinal solubility and, thereby, absorption [37]. Due to its lipid solubility, ivermectin potentially accumulates in fatty tissue that act as reservoir, and is released very slowly over a long time period [36, 38]. In populations with a high prevalence of malnutrition, the high protein binding would result in higher concentrations of free ivermectin, resulting in an increased drug effect and a higher risk of toxicity. However, so far it is not known whether metabolites contribute to the activity and are responsible for the observed “post-ivermectin” effect [4, 11, 39, 40]. Here, mosquitocidal activity is seen even when concentrations of ivermectin drop below relevant LC_50_ values, for example, even at 28 days post-dosing in the IVERMAL trial [11]. This is further supported by mosquito feeding experiments showing that blood meals from human treated with ivermectin have greater mosquitocidal activity than those spiked with pure ivermectin at similar concentrations [40].

Zeng et al. identified the structure of nine ivermectin metabolites in the presence of human liver microsomes, namely 3″-O-desmethyl-H_2_B_1a_ (M1), 4-hydroxy-H_2_B_1a_ (M2), 26-hydroxy-H_2_B_1a_ (M3), 3″-O-desmethyl, 4-hydroxy-H_2_B_1a_ (M4), 24-hydroxy-H_2_B_1a_ monosaccharide (M5), 3″-O-desmethyl, 26-hydroxy-H_2_B_1a_ (M6), 26-hydroxy-H_2_B_1a_ monosaccharide (M7), 4, 26-dihydroxy- H_2_B_1a_ (M8), and 24-hydroxy-H_2_B_1a_ (M9) [41]. Cytochrome P_450_ 3A4 (CYP3A4) is the predominant isoform responsible for the metabolism of ivermectin by human liver microsomes. In brief, ivermectin can be O-demethylated at the disaccharide moiety, undergo deglycosylation, and can be hydroxylated at the aglycone portion. The hydroxylation takes place at the hexahydrobenzofuran, the spiroketal portion of the molecule or both. Tipthara et al. confirmed these results and identified four additional metabolites including ketone and carboxy formation [42].

This study investigated whether metabolites may contribute to the activity of ivermectin against *An. stephensi*. Since no reference standards for ivermectin metabolites were available, a method was set up to produce and purify nine different metabolites. Furthermore, a screening assay was established to estimate whether the metabolites are active against *An. stephensi* at levels observed in humans.

First, an in vitro system was developed using recombinant CYP3A4 and 3A5 isoforms to produce ivermectin metabolites, which were then purified and enriched by semi-preparative high-pressure liquid chromatography (HPLC). Secondly, we studied the pharmacokinetic properties of those metabolites in healthy volunteers who received a single oral dose of 12 mg ivermectin [25]. Finally, blank human blood was spiked with each metabolite fraction at levels matching the maximal signal intensity observed in blood of the pharmacokinetic (PK) study participants. These samples were fed to *An. stephensi* mosquitoes to assess the mosquitocidal activity over 72 h.

## Methods

### Chemicals, reagents and reference compounds

Ivermectin and ivermectin-d_2_ were products of Toronto Research Chemicals (Toronto, Canada). Gradient grade water and methanol as well as formic acid (98–100%) were purchased from Merck (Darmstadt, Germany). Dimethyl sulfoxide (DMSO), ketoconazole, ammonium formate (eluent additive for liquid chromatography–mass spectrometry, LC–MS), bovine serum albumin (BSA), potassium phosphate monobasic (1 M KH_2_PO_4_), and potassium phosphate dibasic (1 M K_2_HPO_4_) were obtained from Sigma-Aldrich (St. Louis, MO, USA). Recombinant human (rh) CYP3A4 supersomes, rh CYP3A5 supersomes, nicotinamide adenine dinucleotide phosphate (NADPH) regenerating solution A (26 mM NADP+, 66 mM glucose-6-phosphate, and 66 mM MgCl2 in H_2_O) and solution B (40 U/mL glucose-6-phosphate dehydrogenase, in 5 mM sodium citrate) were purchased from Corning Life Sciences B.V. (Amsterdam, The Netherlands). Drug-free human blood, stabilised by citrate–phosphate-derivative with adenine was acquired from the local blood donation centre (Basel, Switzerland).

### Metabolism of ivermectin by CYP3A4 and CYP3A5

Ivermectin (10 μM) was incubated in the presence of rh CYP3A4 and rh CYP3A5 supersomes. Production of nine ivermectin metabolites namely desmethyl-H_2_B_1a_ (M1), hydroxy-H_2_B_1a_ (M2), hydroxy-H_2_B_1a_ (M3), desmethyl, hydroxy-H_2_B_1a_ (M4), hydroxy-H_2_B_1a_ monosaccharide (M5), desmethyl, hydroxy-H_2_B_1a_ (M6), hydroxy-H_2_B_1a_ monosaccharide (M7), dihydroxy- H_2_B_1a_ (M8), and hydroxy-H_2_B_1a_ (M9) was evaluated by high-performance liquid chromatography tandem mass spectrometry (LC–MS/MS) based on mass transitions published by Zeng et al. [41]. The reaction mixture of metabolism assays contained 435 μL potassium phosphate buffer (pH 7.4), 25 μL rh CYP3A4 or 3A5 supersomes (1 nmol/mL), 25 μL NADPH solution A, 5 μL NADPH solution B, and 5 μL ivermectin (1 mM). The concentration of ivermectin in the assay mixture corresponded to 10 µM. Ketoconazole was used as CYP inhibitor for both enzyme isoforms. In the case of the inhibition assays, the reaction mixture of the metabolism assay was additionally supplemented with 5 µL ketoconazole (0.1 mM) to receive a final concentration of 1 μM. The assay mixture was heated for 10 min at 37 °C in a Thermomixer 5436 (Eppendorf, Hamburg, Germany) and the reaction was initiated by the addition of supersomes. Samples (50 μL) were taken after 0, 15, 30, 45, and 60 min. The metabolic reaction was stopped by mixing the samples with 150 μL methanol containing 50 ng/mL of ivermectin-d_2_, which was used as internal standard (ISTD solution). After centrifugation (30 min at 3220×*g* and 15 °C, 5810 R Eppendorf centrifuge), sample supernatants (10 µL) were analysed by LC–MS/MS. Metabolite production was quantified by dividing the metabolite peak area with the ISTD peak area. Each metabolism assay was performed in triplicate.

### Production of ivermectin metabolites

Ivermectin (10 μM) was incubated in the presence of human recombinant CYP3A4 and CYP3A5 supersomes. Multiple reactions were prepared for each CYP isoform as described above. The 500 µL reactions were stopped after 2 h of incubation by the addition of 1.5 mL methanol. Samples were vigorously mixed for 30 min and centrifuged (5810 R, Eppendorf) for 30 min at 3220×*g* and 15 °C. Supernatants of all reactions were combined and evaporated at 40 °C for about 4 h using a TurboVap LV evaporator (Caliper Life Sciences, MA, USA). The sample was concentrated by resuspending the residuals in 1 mL methanol. Overall, 10 µL of the sample was injected repetitively (about 50 times) to purify the ivermectin metabolites by semi-preparative high-performance liquid chromatography (HPLC). Nine metabolite fractions were collected, which were evaporated to dryness and resuspended in 1 mL of methanol (Additional file 1: Fig. S1). Details about employed analytical column and HPLC gradient programme are given in Additional file 1: Table S2. The time-intervals of the fractions are summarised in Additional file 1: Table S4. The fractions were aliquoted and kept at − 20 °C until used for *An. stephensi* assays.

### Clinical pharmacokinetics of ivermectin metabolites

The signal intensity (peak height) time course of nine ivermectin metabolites (M1–M9) were recorded in blood samples originating from a clinical trial [25]. In brief, twelve healthy volunteers (6 males, 6 females) received a single oral dose of 12 mg ivermectin (4 tablets Stromectol® 3 mg, MSD, Courbevoie Cedex, France). Peripheral venous blood samples were collected before dosing and after 1, 2, 4, 6, 8, 12, 24, 48 and 72 h. Blood samples (50 µL) were extracted by the addition of 150 µL ISTD solution. Samples were mixed for 1 min and centrifuged for 30 min at 3220×*g* and 15 °C (5810 R centrifuge). Twenty microlitres of supernatant were injected into the LC–MS/MS system to determine the signal intensity of the metabolites.

### LC–MS/MS analysis of ivermectin metabolites

Ivermectin and its metabolites were analysed by LC–MS/MS based on a previously validated bioanalytical method [43]. The method was modified for the detection and quantification of nine ivermectin metabolites based on the study of Zeng et al. [41]. The different LC–MS/MS settings were applied for the metabolism, pharmacokinetic and metabolite fractioning assays (Additional file 1: Table S1, S2, S3). All analyses were performed on a modular HPLC (Shimadzu, Kyoto, Japan) composed of four pumps (2 × LC-30AD, 2 × LC-20AD), an autosampler (SIL 30-AC MP), a system controller (CBM-20A), two degassing units (DGU-20A5 and DGU-20A3R), a column oven (CTO-20AC), a low-pressure valve (FCV-12AH), and a fraction collector (FRC-12A). The HPLC system was connected to an API 5000 triple quadrupole mass spectrometer (AB Sciex, Ontario, Canada) that was equipped with a turbo electrospray ionization source. The system was operated with Analyst Software 1.7 (AB Sciex) and acquired data were analysed using MultiQuant Software 3.0.3 (AB Sciex).

Zeng et al. reported on the structure of nine ivermectin metabolites and published the corresponding mass transitions [41]. These mass transitions were integrated into the method. Ivermectin and its metabolites were analysed by multiple reaction monitoring in the positive mode. The applied mass transitions and analyte specific settings are given in Additional file 1: Table S1. Nitrogen was used as collision gas (4 psi), curtain gas (20 psi), ion source gas I (60 psi) and II (50 psi). Ion spray voltage was set to 5500 V and the source temperature to 300 °C.

A Kinetex C8 analytical column (50 × 2.1 mm, 2.6 µm, 100 Å, Phenomenex, CA, USA) was used for pharmacokinetic assays. A Luna C8(2) column (150 × 2.0 mm, 5 µm, Phenomenex) was employed for the metabolism assays and fractioning of the ivermectin metabolites. Mobile phase A was an aqueous solution of 20 mM ammonium formate with 0.1% formic acid. Methanol plus 0.1% formic acid was used as mobile phase B. Chromatography was performed at 55 °C. The dual binary flow programmes used for the different assays are summarised in Additional file 1: Tables S2 and S3.

Metabolite fractions were isolated with a FRC-12A fraction collector that was coupled to the HPLC system. LabSolutions software 5.97 (Shimadzu) was employed to operate the fractioning. The following nine fractions (F1-F9) were collected: F1; 4.50–4.85 min, F2; 4.90–5.15 min, F3; 5.15–5.50 min, F4; 5.50–5.70 min, F5; 5.75–6.00 min, F6; 6.10–6.40 min, F7; 6.50–6.75 min, F8; 6.95–7.35 min, and F9; 7.40–7.80 min. Metabolite M7 was predominantly present in F1, M8 in F2, M6 in F3, M3 in F4, M5 in F5, M9 in F6, M4 in F7, M2 in F8, and M1 in F9. Metabolite composition of the fractions was assessed by LC–MS/MS so as to quantify carry-over effects and the purity of each fraction. Additional file 1: Fig. S1 depicts the chromatogram of the metabolites before and after fractioning. Most fractions contained also the metabolite(s) of the previous fraction due to carry-over effects (e.g. M7 is present in fraction 1 and 2).

### Pharmacokinetic analysis

The pharmacokinetic parameters of ivermectin metabolites in twelve healthy volunteers were calculated in the non-compartmental analysis (NCA) framework of PKanalix (version 2021R2, http://www.lixoft.com, Antony, France), for the most abundant metabolites. The integral method “linear up log down” was used on area/internal standard area ratio. Metabolites were included when peak height at C_max_ was above 500 counts per second (cps), hence M5, M7, M8 and M9 were excluded from the NCA analysis.

### *Anopheles stephensi* mosquitoes bioassays

#### Mosquito rearing

*Anopheles stephensi* strains were reared according to standard conditions [44] under constant temperature (27 °C ± 2 °C) and relative humidity (70% ± 10%), at a cycle of 12:12 h in light/darkness. To allow for mating, female and male mosquitoes were kept in the same cage. Females were membrane-fed with fresh pig blood once a week. Mosquito eggs were harvested on round filter papers (⌀ 8 cm, Sartorius, Göttingen, Germany) placed on top of a moist sponge. The eggs were left to sclerotise and to dry for seven days in the insectary, and hatched by placing a slice of the egg-coated filter paper in a glass dish filled with tap water treated with 0.012% (v/v) AquaSafe (Aquasafe, Seevetal, Germany). About 300 larvae were transferred into white plastic trays containing 600 mL AquaSafe-treated water. The larvae were grown at 27 °C in an incubator (Aqualytic, Dortmund, Germany) and fed daily with TetraMin® fish food (Tetra, Melle, Germany). Once the larvae reached the 4th instar, they were transferred into emergence cages (375 mm × 445 mm × 495 mm) and adults were provided with 10% aqueous sucrose solution ad libitum.

#### Blood meal preparation

The required ivermectin metabolite fraction volume was calculated according to the ratio of the mean peak area of the metabolite measured in the blood of participants treated with ivermectin and the mean intensity of the metabolite measured in blank blood (T0 PK sample) that was spiked with a known amount of the metabolite fraction. In brief, an aliquot of 10 µL of each of the nine ivermectin metabolite fractions was evaporated and resuspended in 50 µL of blank blood. In total, three T0 blank blood samples originating from different PK study participants were used to resuspend the metabolites (Additional file 1: Table S5) [25]. The signal intensity was recorded for every metabolite by extracting the 50 µL blood with 150 µL of ISTD solution. The metabolite peak area was determined by LC–MS/MS (see section LC-MS/MS analysis of ivermectin). Moreover, the PK samples of all study participants were equally extracted and analysed to derive the maximal blood level (C_max_) from the blood level-time curves. Afterwards, the mean metabolite peak area of the spiked blank blood was compared with the mean peak area of the C_max_ samples (Additional file 1: Table S5). The amount of each metabolite fraction was either reduced or increased to match the peak area of C_max_ samples. If the metabolite was not detectable in PK samples, the amount of metabolite was adjusted to the lower limit of detection of the method. In this case, blank blood was spiked with the metabolite fraction to receive a signal intensity that is three times larger than the corresponding noise level.

On the day of the treatment, each of the nine ivermectin metabolite fractions were evaporated and resuspended in 3 mL blank human blood to receive metabolite levels that matched C_max_ levels observed in PK samples (Additional file 1: Table S5). As a positive control, ivermectin was mixed with blank blood to obtain a final concentration of 50 ng/mL. This concentration corresponds to the C_max_ of ivermectin following an oral dose of 12 mg ivermectin [25]. The final DMSO content in blood was 0.1%, which was also the DMSO concentration used for the negative control assays (0.1% DMSO in blank blood). Inclusion of blank fractions as additional negative controls were not considered, because it would not have been feasible to also test the corresponding blank fraction for each metabolite fraction (F1–F9). The blood preparations were rotated at room temperature for 20 min to ensure a uniform metabolite distribution (Rotator Genie, Scientific Industries, Bohemia, USA). The dissolution of the metabolites was confirmed by LC–MS/MS.

#### Mosquito preparation and membrane feeding procedure

Around 50–70 female mosquitoes aged 5–10 days post-emergence were transferred with a mouth aspirator into 20 mL paper cups. A piece of mosquito netting was used to cover the cups. Mosquitoes were starved for 24 h before treatment. The mosquito blood meals were heated in a water bath (SAHARA PPO S5P Heated Bath Circulators, Thermo Fisher Scientific, New Hampshire, USA) for 20 min at 39 °C. A Petri dish (35 mm × 10 mm, BD, Franklin Lakes, USA) was filled with 3 mL of warm blood, sealed with a piece of sealing film (Parafilm®, Huberlab AG, Aesch, Switzerland). The Petri dish was placed upside down on top of the net sealing the paper cup. Female mosquitoes were allowed to feed for 20 min on the blood through the net and the sealing film. The feeding was conducted in a climate chamber (HPP110, Memmert GmbH + Co.KG, Schwabach, Germany) at 27 °C, 70% humidity, and 30% light intensity. After feeding, the cups were put on ice to immobilise the mosquitoes and only the fully engorged mosquitoes were selected for the assay. The mosquitoes were returned to the climate chamber to assess their activity and survival for 3 days.

#### Metabolite effect on *Anopheles stephensi* survival

In the framework of screening assays, mosquitoes were treated with blood containing each metabolite corresponding to C_max_ levels observed in PK samples. Afterwards, the LC_50_ was estimated for the most active metabolite fractions in three independent assays. The mortality effect of the nine metabolite fractions, ivermectin (positive control), and blank blood (negative control) on mosquito survival and activity was assessed at 24 h, 48 h and 72 h post-feeding. In screening assays, eleven treatments were evaluated with three replicates using three batches of 50–70 mosquitoes. At each time point, dead and alive mosquitoes were counted and their activity was rated with the following scores: + 2; the mosquito is flying and resides mainly on the top of the paper cup, + 1; the mosquito is moving but stays on the bottom of the paper cup, 0; the mosquito is classified as dead and does not move upon physical contact.

The concentration of compound required to kill 50% of adult mosquitoes in 3 days (lethal concentration 50%, 3-day LC_50_) was evaluated for ivermectin and the most active metabolites M1 and M2. Therefore, three replicates using three batches of 50–70 mosquitoes were treated with different dilutions of ivermectin (^1^/_4_ C_max_, ^1^/_6_ C_max_, ^1^/_10_ C_max_, ^1^/_20_ C_max_, ^1^/_50_ C_max_), M1 (C_max_, ^1^/_2_ C_max_, ^1^/_4_ C_max_, ^1^/_6_ C_max_, ^1^/_10_ C_max_) and M2 (C_max_, ^1^/_2_ C_max_, ^1^/_3_ C_max_, ^1^/_4_ C_max_, ^1^/_5_ C_max_). A pilot test was performed to evaluate optimal dilutions  matching the expected LC_50_ values (data not shown). A C_max_ of 50 ng/mL was used for ivermectin [25], consequently the LC_50_ was evaluated using ivermectin concentrations ranging between 1 and 12.5 ng/mL.

### Statistical analysis

Student's t test was adopted to test the significance of all mean survival differences between groups, using the R package *ggsignif* (version 0.6.4). Survival analyses, summary and visualisation of survival analysis were computed using the GNU R packages *survival* (version 3.1-8) and *survminer* (version 0.4.9). Replicates were pooled and analysed by the logrank test and the hazard ratio with 95% confidence intervals. LC_50_ in *An. stephensi* mosquito were approximated with regression analysis, using GNU R package *drc* [45] (version 3.0-1) for analysis of dose–response curves (Additional file 1: Fig. S3). The time above LC_50_ estimation (Additional file 1: Fig. S4) was computed using the GNU R package *data.table* (version 1.14.6).

### Software

Data analysis and visualisation were performed with GNU R (version 4.2.2, R Foundation for Statistical Computing, http://www.R-project.org, Vienna, Austria), GraphPad Prism (version 9.3.1, San Diego, USA), and Adobe Illustrator (version 26.2.1, San José, USA).

## Results

### Ivermectin metabolism experiments

**Fig. 1:**
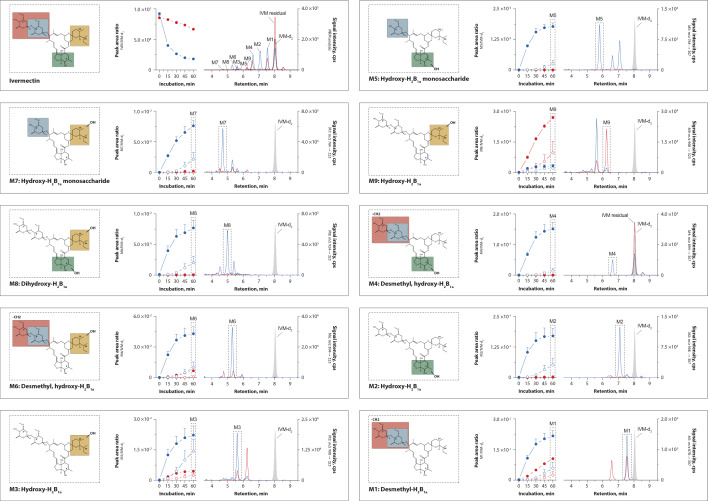
Ivermectin metabolism. Chemical structure of ivermectin and putative structure of the metabolites (M1–M9): M1: Desmethyl-H_2_B_1a_, M2: Hydroxy-H_2_B_1a_, M3: Hydroxy-H_2_B_1a_, M4: Desmethyl, hydroxy-H_2_B_1a_, M5: Hydroxy-H_2_B_1a_ monosaccharide, M6: Desmethyl, hydroxy-H_2_B_1a_, M7: Hydroxy-H_2_B_1a_ monosaccharide, M8: Dihydroxy-H_2_B_1a_, M9: Hydroxy-H_2_B_1a_. Ivermectin (10 µM) was incubated for 60 min in the presence of CYP3A4 (blue) or CYP3A5 (red) enzymes (supersomes). Incubations were performed in the presence (empty circle) or absence (filled circle) of ketoconazole (1 µM), a potent 3A inhibitor. Formation of the ivermectin metabolites is shown as mean peak area ratio (analyte peak area: ivermectin-d_2_ area). The error bars correspond to the standard error of the mean. A representative chromatogram recorded 60 min post-incubation is depict for each metabolite transition. Ivermectin-d_2_ (IVM-d_2_) was added as point of comparison into each chromatogram

Ivermectin was incubated with recombinant CYP3A4 and 3A5 to produce the nine metabolites, then their signal intensity was recorded by LC–MS/MS. Ivermectin depletion, metabolite formation, putative chemical structure, and representative chromatograms are depicted in Fig. 1.

CYP3A4 metabolised 80% of ivermectin within 1 h, while in the same time only 20% of ivermectin was metabolised by CYP3A5. In the presence of CYP3A4, maximum peak intensities were observed for M1, M2, and M4. However, the peak intensity of different compounds does not necessarily correlate with the actual quantity. All metabolites, except M9, were more readily produced by CYP3A4 than by CYP3A5. In fact, M9 production was very effective with CYP3A5, while CYP3A4 formed only minor amounts. In addition, M9 formation by CYP3A5, although not by CYP3A4, could be substantially inhibited by the addition of ketoconazole. In contrast, ketoconazole clearly inhibited the formation of all other metabolites by CYP3A4, verifying the validity of the metabolic assay. In summary, CYP3A4 is mainly responsible for the metabolism of ivermectin, yet CYP3A5 is required to produce M9.

### Clinical pharmacokinetics of ivermectin metabolites

**Fig. 2:**
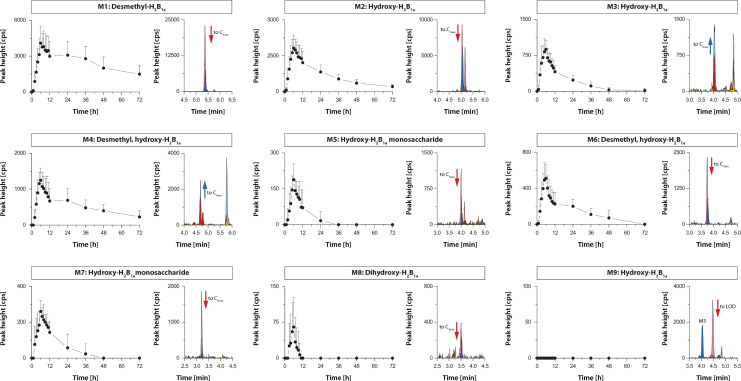
Ivermectin metabolites pharmacokinetics in human. Mean blood level-time curves of ivermectin metabolites determined in pharmacokinetic (PK) study samples. A number of 12 individuals received a single oral dose of 12 mg ivermectin. The blood samples of the participants were analysed to derive the maximal blood level (C_max_) from the metabolite peak intensity time plots. The error bars correspond to the standard deviation. The C_max_ (blue) and T0 (yellow) chromatograms of PK subject 6 are shown next to the blood level-time curves. The metabolite signals of spiked blank blood samples (T0 sample of PK subject 6) are colored in red. The arrow in the chromatogram indicates whether the amount of the spiked sample had to be increased (red) or decreased (blue) to reach the peak intensity of C_max_ samples. If a metabolite was not detected in PK samples, as for M9, the metabolite level was adjusted to the lower limit of detection of the method. In this case, blank blood fed to mosquitoes was spiked with the metabolite to obtain a signal intensity three times greater than the corresponding noise signal. It should be noted that the peak area of M4 in spiked blood samples was on average larger than the observed C_max_ levels. However, the M4 peak area of the spiked blank sample of subject 6 was smaller than the observed C_max_. For this reason, the amount of M4 was reduced to obtain values that corresponded to the average C_max_ of all 12 study participants (Additional file 1: Table S5)

The pharmacokinetics of nine ivermectin metabolites, M1–M9, were assessed in blood samples of twelve healthy volunteers who received a single oral dose of 12 mg ivermectin [25]. The mean peak intensity time curves of the ivermectin metabolites are shown in Fig. 2. All metabolites could be detected apart from M9, showing a profile as expected for an oral drug administration. The pharmacokinetic parameters are summarised in Table 1. Maximal peak levels of the metabolites were observed later on average (T_max_ range: 5.4 h–7.0 h) than recorded for ivermectin (T_max_: 4.4 h). An average terminal elimination half-life of 38.4 h (T_½_ range: 27.1 h–57.5 h) was calculated for the metabolites. This is close to the half-life of ivermectin (T_½_: 38.9 h) determined in the same subjects [25]. Similar to the in vitro metabolism assay, largest peaks were observed for M1 > M2 > M4 > M3, while the peak intensities of the other metabolites were rather low. For the metabolites, the mean residence time from the time of dosing to the time of the last measurable concentration (MRT_last_) was 24.2 h (MRT_last_ range: 13.9 h–33.8 h). The measurement of the maximal metabolite peak intensities in PK samples permitted the preparation of the blood samples with equal amounts of metabolite. Consequently, blood samples were produced with a pharmacologically relevant amount of metabolite, as observed after administration of a regular ivermectin dose. An absolute quantitative determination was not possible as no ivermectin metabolite reference standards were commercially available, and none could be synthesised in-house due to the structural complexity and by consequence very involved chemical synthetic process.

**Table 1:** Pharmacokinetic parameters of ivermectin metabolites in whole blood for 12 subjects after a dose of 12 mg ivermectin

Ivermectin metabolite	T_max_ [h]	C_max_ [ratio]	Half-life [h]	MRT_last_ [h]
M1	7.0 ± 0.5	0.011 ± 0.001	54.2 ± 4.7	33.8 ± 0.9
M2	6.3 ± 0.3	0.010 ± 0.0010	31.9 ± 3.4	25.8 ± 0.7
M3	5.3 ± 0.2	0.003 ± 0.0003	15.9 ± 2.8	13.9 ± 1.3
M4	6.0 ± 0.2	0.004 ± 0.0004	57.5 ± 13.2	29.4 ± 1.7
M5	6.4 ± 0.4	N/A	N/A	N/A
M6	5.4 ± 0.4	0.002 ± 0.0002	32.4 ± 4.1	18.3 ± 1.6
M7	6.1 ± 0.4	N/A	N/A	N/A
M8	5.0 ± 0.3	N/A	N/A	N/A
M9	6.0 ± 0.8	N/A	N/A	N/A

Parent	T_max_ [h]	C_max_ [ng/mL]	Half-life [h]	
Ivermectin*	4.4 ± 1.4	70.7 ± 16.1	38.9 ± 20.8	

Blood was collected on volunteers pre- and up to 72 h post-ivermectin dose (Duthaler et al., 2019) [25]. Non-compartmental analysis (NCA) was used to derive the ivermectin metabolites pharmacokinetic parameters. Pharmacokinetic parameters are showed as mean (± SE). The ratio area/internal standard area was used for the metabolites, whereas ivermectin quantitative data with SD was taken in plasma (*) from [25] . The least abundant metabolites M5, M7, M8 and M9 were excluded from the NCA analysis. Abbreviations: standard error (SE); time of peak concentration (T_max_); peak concentration (C_max_); Mean residence time from the time of dosing to the time of the last measurable concentration for a substance administered by extravascular dosing (MRT_last_)

### Production and purification of ivermectin metabolites

Both CYP isoforms were required to produce sufficient amount of all nine metabolites, considering that M9 was mainly produced by CYP3A5. The metabolites were purified by semi-preparative HPLC collected in nine fractions (Additional file 1: Fig. S1). The metabolite composition of the different fractions are summarised in Additional file 1: Table S4. In brief, fraction 1 contained mainly M7 (m/z 764 → 323). An additional minor peak was detected corresponding to the mass transition of M6 (m/z 894 → 323), however the compound eluted earlier than M6 (4.68 vs. 5.24 min). This peak corresponds most likely to another unidentified ivermectin metabolite, since it was formed by CYP 3A5 and its formation could be inhibited by the addition of ketoconazole (data not shown). Fraction 2 contained mainly metabolite M8, but also approximately 24% carry-over of fraction 1. M6 was the main metabolite in fraction 3 containing a carry-over of about 21% from fraction 2. Interestingly, a peak corresponding to the mass transition (m/z 764 → 323) of M7 was also present in fraction 3. This peak exhibited another retention time than M7 and eluted at the same time as M6. Hence, it is likely that M6 is insource fragmented to M7 by being deglycosylated during the ionization process. M3 is equally present in fraction 4 and 5. However, fraction 4 also contains M6 (35% carry-over) and fraction 5 displays largest amounts of M5. Fraction 6 exhibits primarily M9 and 29% carry-over of M5 originating from fraction 5. M4 was for the most part present in fraction 7 but also largely in fraction 8. In fraction 7, an M1 signal (m/z 878 → 307) was detected at the same retention time as of M4 (m/z 894 → 307), possibly induced by dehydroxylation of M4 into M1 during ionization. Fraction 8 contained besides M4 mainly M2. Finally, M1 was collected in F9, which contained a 13% carry-over of fraction 8. In summary, fraction 1 was used to spike blank blood with M7, fraction 2 for M8, fraction 3 for M6, fraction 4 for M3, fraction 5 for M5, fraction 6 for M9, fraction 7 for M4, fraction 8 for M2, and fraction 9 for M1.

### Activity of ivermectin metabolites against *Anopheles* mosquito

**Fig. 3:**
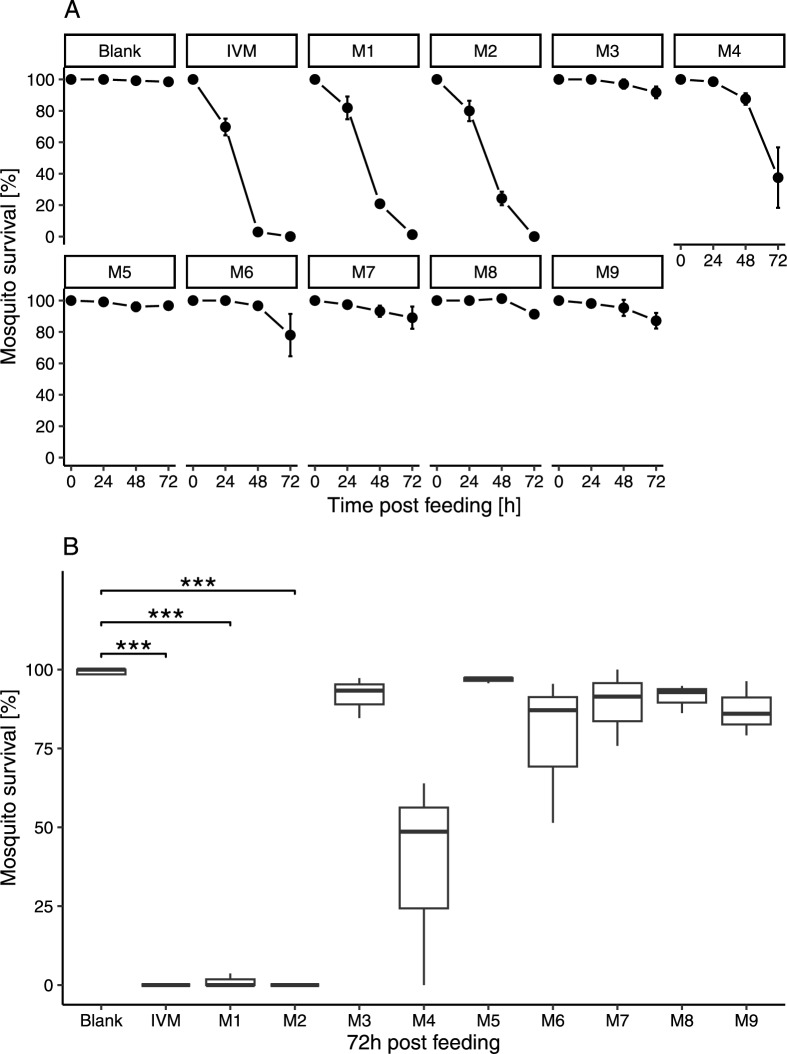
Screening for ivermectin metabolites effect on mosquito mortality. *Anopheles stephensi* mosquitoes were treated with blank human blood, blood containing ivermectin (IVM, 50 ng/mL), or ivermectin metabolite (M1-M9). Data represent three independent replicates per compound, each containing an average of 28 mosquitoes/condition. **A** Mortality over time. Mean mosquito survival was assessed after 24 h, 48 h and 72 h. Error bars correspond to the standard error or the mean. **B** Percent survivorship of *An. stephensi* 72 h; after feeding on ivermectin or its metabolites (M1–M9). Significant differences in the means of two groups are marked, *** shows a p-value < 0.001 with a t-test

**Fig. 4:**
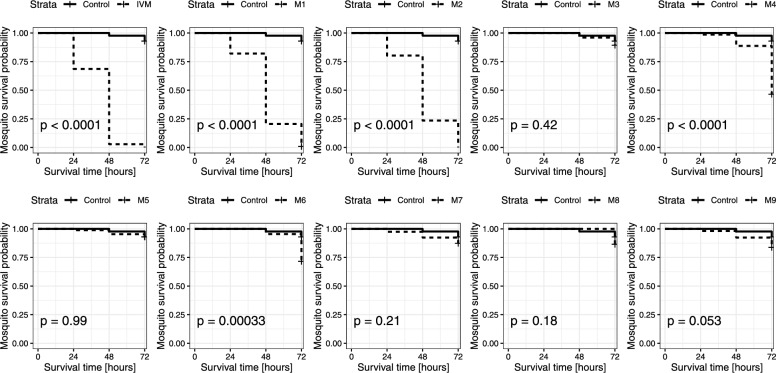
*Anopheles stephensi* survival probability after imbibing blood meals containing ivermectin and ivermectin metabolites. *Anopheles stephensi* mosquitoes were given a blood meal that contained ivermectin (IVM) or its metabolites (M1–M9) levels corresponding to those interpolated from the IVM pharmacokinetic curve. The mosquitoes’ survival was monitored at 24 h, 48 h and 72 h post feeding. Ivermectin (H_2_B_1a_) metabolites: M1: Desmethyl-H_2_B_1a_, M2: Hydroxy-H_2_B_1a_, M3: Hydroxy-H_2_B_1a_, M4: Desmethyl, hydroxy-H_2_B_1a_, M5: Hydroxy-H_2_B_1a_ monosaccharide, M6: Desmethyl, hydroxy-H_2_B_1a_, M7: Hydroxy-H_2_B_1a_ monosaccharide, M8: Dihydroxy-H_2_B_1a_, M9: Hydroxy-H_2_B_1a_. Mosquitoes that exhibited significantly reduced survival (Log rank test) compared to control (blood meal contained only dimethyl sulfoxide) have a p-value < 0.0001

The nine isolated metabolites were spiked to human whole blood matching peak intensities observed in the clinical pharmacokinetics study samples (Fig. 2). Mosquito mortality (Figs. 3, 4) and activity (Additional file 1: Fig. S2) were recorded as entomological measures for the effect of ivermectin and its metabolites. In screening assays, eleven treatments were evaluated with three replicates using three batches of mosquitoes (mean of 28 mosquitoes/treatment, minimum of 11 and maximum of 50). The mean survival of mosquitoes after a blood meal containing ivermectin, M1, M2, M4, and M6 is decreased (Fig. 3, Fig. 4). The ivermectin positive control killed 100% of mosquitoes 72 h after treatment, similar to M1 with 98.8% of mosquitoes dead after 72 h, and 100% for M2. In the negative control (blank blood), 98.5% of mosquitoes survived after 72 h. Significant differences were found in the median survival of mosquitoes 72 h post-treatment with M1 (p < 0.001) and M2 (p < 0.001). The activity of mosquitoes is reduced in a similar way compared to their mortality, as reflected by their activity score (Additional file 1: Fig. S2). Mosquitoes were not able to fly 24 h after ingesting ivermectin (0.71 ± 0.05), M1 (1.07 ± 0.04) or M2 (1.20 ± 0.12), and after 48 h most mosquitoes were not moving, as their scores approached zero, with ivermectin (0.03 ± 0.02), M1 (0.23 ± 0.02) and M2 (0.28 ± 0.05). Ivermectin and its metabolites M1, M2 and to a lesser extent M4 and M6, increase the mortality and decrease the activity of *Anopheles* mosquitoes that ingest it in a blood meal.

**Fig. 5:**
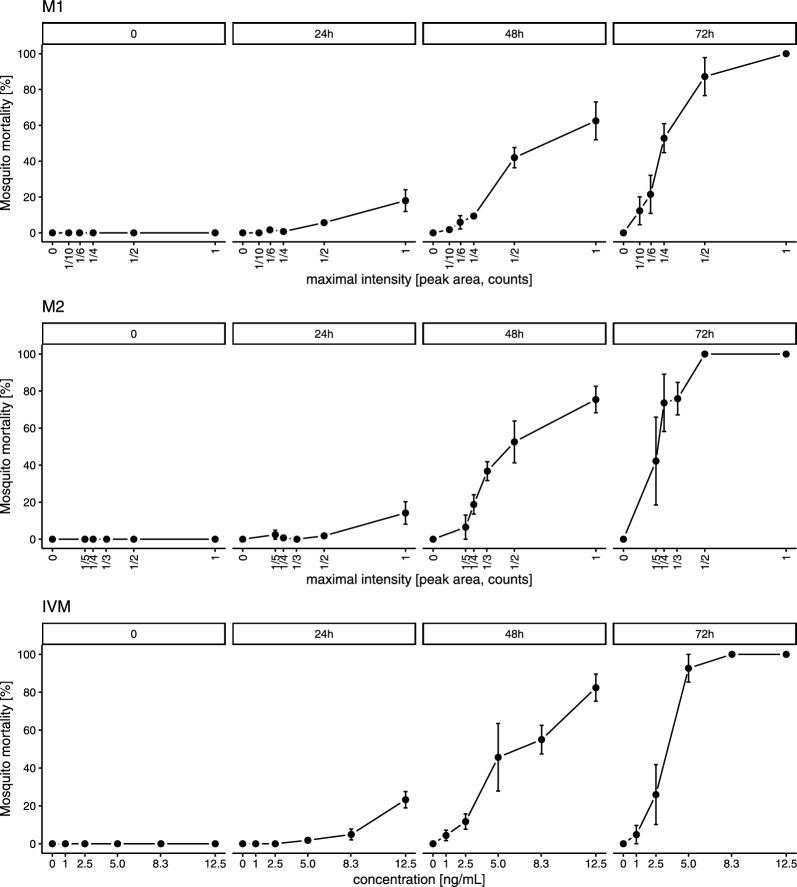
Ivermectin metabolites effect on mosquito mortality: LC_50_. Multiple amounts of ivermectin (IVM), desmethyl-H_2_B_1a_ (M1) or hydroxy-H_2_B_1a_ (M2) were fed to mosquitoes to determine the lethal concentration that killed 50% of the mosquitoes (LC_50_) 72 h after treatment. The blood samples of the participants of the pharmacokinetics trial [25] were analysed to derive the maximal blood level (C_max_) for ivermectin, M1 and M2 (Fig. 2). An administration of 12 mg ivermectin yielded a C_max_ of 50 ng/mL for ivermectin, therefore the LC_50_ was evaluated using ivermectin concentrations ranging between 1 and 12.5 ng/mL. For the metabolites M1 and M2, C_max_ corresponds to the maximal intensity (peak area, counts) from the metabolite peak intensity time plots. *Anopheles stephensi* were treated with different dilutions of ivermectin (^1^/_4_ C_max_, ^1^/_6_ C_max_, ^1^/_10_ C_max_, ^1^/_20_ C_max_, ^1^/_50_ C_max_), M1 (C_max_, ^1^/_2_ C_max_, ^1^/_4_ C_max_, ^1^/_6_ C_max_, ^1^/_10_ C_max_) and M2 (C_max_, ^1^/_2_ C_max_, ^1^/_3_ C_max_, ^1^/_4_ C_max_, ^1^/_5_ C_max_). Mean mosquito survival was assessed after 24, 48 and 72 h. Error bars correspond to the standard error of the mean

A common metric of the killing efficacy of ivermectin in mosquitoes is the lethal concentration 50% (LC_50_), i.e. the concentration at which 50% of mosquitoes die over a defined observation period. Multiple doses/concentrations of compound were orally fed to mosquitoes to determine their LC_50_ in *An. stephensi* from 24 to 72 h after treatment. The experiment was performed in triplicate, with an average of 24 mosquitoes per compound dilution. A total number of 485, 516 and 424 mosquitoes were fed for ivermectin, M1 and M2. The 1–2–3-day LC_50_ of ivermectin and of the most promising candidates, M1 and M2, were investigated in *An. stephensi* (Fig. 5). The 3-day-LC_50_ of ivermectin, M1 and M2 are presented in Table 2, derived from the nonlinear model presented in Additional file 1: Fig. S3. When taken together with the pharmacokinetics data from the volunteers after a single oral dose of 12 mg ivermectin (Fig. 2 and Table 2), the metabolites levels above their respective 3-day-LC_50_ values last 69 h for M1 and 34 h for M2, compared to 69.3 h for ivermectin (Additional file 1: Fig. S4).

**Table 2:** Lethal concentrations (LC_50_) of ivermectin metabolites for *Anopheles stephensi* at day 3 post-blood feeding

	3-day LC_50_	95% confidence intervals
M1 [C_max_]	0.25	0.18–0.32
M2 [C_max_]	0.21	0.16–0.26
Ivermectin [ng/mL]	3.07	2.49–3.66

Multiple concentrations of ivermectin , desmethyl-H_2_B_1a_ (M1) or hydroxy-H_2_B_1a_ (M2) were fed to mosquitoes to determine the lethal concentration that killed 50% of the mosquitoes (LC_50_) 72 h after treatment. For M1 and M2, C_max_ corresponds to the maximal intensity (peak area, count) in blood observed in human pharmacokinetics study samples, whereas for ivermectin it corresponds to the maximal blood concentration of 50 ng/mL

## Discussion

The clinical applications of ivermectin are remarkably broad. This widely used drug can treat not only veterinary infections but also human ones, caused by various endo- and ectoparasites, as well as rosacea skin conditions [46]. Nonetheless, the pharmacokinetics and therapeutic role of ivermectin metabolites has not yet been elucidated.

This study set out to develop a protocol to produce and isolate nine different ivermectin metabolites. It also shows that CYP3A4 and CYP3A5 metabolise ivermectin, while CYP3A4 readily produces all metabolites except M9 (Hydroxy-H_2_B_1a_)—that is mainly formed by CYP3A5. All metabolites apart from M9 are measurable in human blood after a single oral dose of 12 mg ivermectin. Pharmacokinetic analysis reveals that the mean residence time of the metabolites is shorter (MRT_last_ range: 13.9–33.8 h) as reported for ivermectin after a single dose of 150 µg/kg (89.5 h) [47]. Finally, this study demonstrates that ivermectin metabolites M1 and M2 are mosquitocidal at concentrations observed in humans treated with a regular dose of ivermectin, and may contribute to the pharmacological effect of ivermectin treatments.

Zeng et al. have previously reported on the metabolic fate of ivermectin in human in vitro systems, and recently Tipthara et al. published in vitro as well as in vivo data [41, 42]. The present investigation employed a multiple reaction monitoring LC–MS/MS method for nine ivermectin metabolites, derived from the published mass fragmentation data of Zeng et al. This study supports evidence from previous observations made by Zeng et al. that ivermectin is readily metabolised by CYP3A enzymes—as the metabolites were formed in the presence of human recombinant CYP3A4 and CYP3A5—and that the formation could be inhibited by ketoconazole, a selective CYP3A4/5 inhibitor [41, 48]. In addition, CYP2C8 is involved in the hydroxylation of the spiroketal moiety of ivermectin [42]. This current study found that CYP3A5 is involved in the O-demethylation of ivermectin (Fig. 1), as previously described by Tipthara et al. Moreover, another important finding is that CYP3A5 is mainly responsible for the formation of M9 (Hydroxy-H_2_B_1a_) and forms the metabolites M3 and M6—yet to a lesser extent than CYP3A4. The involvement of CYP3A5 in metabolising ivermectin might be of clinical relevance considering that its expression greatly varies between individuals according to their ethnicity and geographical location because of genetic polymorphisms in CYP3A4 and/or CYP3A5 genes [49]. Possibly, pharmacokinetics, treatment response and appearance of adverse drug events might be linked to the patients CYP3A5 genotype. Importantly, single nucleotide polymorphism (SNP) in the CYP3A5 gene alter the expression levels of CYP3A5 enzymes leading to inter-individual differences in the enzyme activity. Racial differences might affect metabolism of and response to ivermectin considering that CYP3A5 is more frequently seen in Africans than Caucasians [49]. Since neither the activity nor the toxicity of ivermectin metabolites have been investigated, the clinical significance of CYP3A5 polymorphisms and drug–drug interactions remain unclear. In light of the greater contribution of CYP3A4 to ivermectin metabolism rather than CYP3A5, it seems unlikely that pharmacogenetic differences between malaria endemic regions would be of importance.

The results of this study show that the most intense signals in the in vitro metabolism assays were recorded for M1 (desmethyl-H_2_B_1a_) and M2 (hydroxy-H_2_B_1a_). However, this does not necessarily indicate that those two metabolites are the most abundant. Yet, assays with radiolabeled ivermectin imply that M1 and M2 are the major in vitro metabolites of ivermectin [41]. Interestingly, M1 was also produced by rat, pig, sheep and dog microsomes, which suggests that the O-demethylation of ivermectin may be also relevant for the treatment of livestock and domestic animals [50–53]. Drug–drug interaction studies were carried out in animals to assess the effect of CYP inhibition and induction on the pharmacokinetics of ivermectin. Co-administration of ivermectin and ketoconazole increased exposure to ivermectin in sheep, but did not reduce the levels of metabolite M1 [54]. The authors concluded that the observed interaction is rather due to inhibition of P-glycoprotein efflux transporters than of CYPs. The same conclusion was drawn by Hugnet et al., since ketoconazole did not decrease M1 levels in dogs but significantly increased exposure to ivermectin [53]. On the contrary, ketoconazole did not alter the PK of ivermectin in invertebrates, most likely because it is rapidly excreted by *Aedes aegypti* mosquitoes [16]. Finally, the CYP activity in rats was increased by administering either rifampicin or phenobarbital daily for 1 week, but still the disposition kinetics was only modified for ivermectin—and not the investigated metabolites [55]. These examples imply that the mechanism of interaction with ivermectin is rather due to interference with P-glycoprotein efflux transporters than with CYPs. Nonetheless, in this study ketoconazole was found to clearly inhibit the metabolism of ivermectin in vitro, and drug–drug interaction studies in humans are needed to assess the impact of CYP inhibition on the response and safety of ivermectin treatments. This is in line with previous studies that showed oral bioavailability of ivermectin being significantly altered in mammals when co-administered with drugs interacting with CYP3A and P-gp, two systems that are frequently co-located and act in synergy because of overlapping substrate affinities [56, 57]. As such they do not only affect overall metabolism and distribution, leading to appreciable changes in metabolite profiles, but also pre-systemic metabolism. Here, CYP3A can reduce concentrations of substrates in gastrointestinal tissue and allow for more efficient efflux via P-gp. Pharmacoenhancement and boosting of ivermectin action and metabolism is, therefore, possible on several levels.

The LC–MS/MS method presented here was able to assess the kinetic disposition of eight ivermectin metabolites over a period of up to 72 h post-treatment in human whole blood. A previous study also detected the most abundant metabolites namely desmethyl-H_2_B_1a_ (M1), hydroxy-H_2_B_1a_ (M2), and desmethyl, hydroxy-H_2_B_1a_ (M4) in human blood samples collected 24 h post-treatment [42]. Only traces of M9 were observed in pharmacokinetic samples, either because the method is not sensitive enough, or the study participants were not expressing sufficient amounts of CYP3A5—as it is frequently observed in Caucasians [58]. Nevertheless, the CYP3A5 polymorphism of the study participants was not assessed, which would have been required to confirm this hypothesis.

The drug concentration in the blood and the time the drug remains in circulation are important factors affecting the pharmacological effect. The results of the clinical pharmacokinetic study in humans indicates that ivermectin metabolites T_max_ (5–7 h) are shifted in time and occur later than ivermectin (4.4 h). Moreover, some metabolites elimination half-lives are longer (15.9–57.5 h) than ivermectin (38.9 h), allowing for an overall longer timeframe when mosquitoes can potentially be killed by certain ivermectin metabolites (M4, M1) than what would be expected from ivermectin alone. As another measure for the length of exposure to a substance, the mean residence times (MRT) of ivermectin metabolites (M1–M6) were calculated by non-compartmental analysis (NCA) of pharmacokinetic profiles (Table 1). The MRT is the average time a molecule resides in the body and reflects absorption and elimination rates. MRT was used as a measure for elimination times for both ivermectin and its metabolites, as this allows comparing the duration of exposure. After a single oral 12 mg ivermectin dose (corresponding to a mean dose of 181 µg/kg), the mean residence time (MRT_last_) is shorter for some of the metabolites (MRT_last_ range: 13.9–33.8 h) compared to ivermectin. A reported MRT of 89.5 h was measured for ivermectin after a single dose of 150 µg/kg ivermectin [47]. Because of limited sampling time the MRT of the compounds may be biased. In this proof-of-concept study with blood concentration measurements until 72 h post-dose of ivermectin, sampling times were chosen based on operational feasibility and empirical reasoning.

Kobylinski et al. demonstrated first evidence that metabolites might contribute to the activity of ivermectin as they observed that ivermectin spiked blood was less mosquitocidal than blood from treated humans with matching ivermectin levels [40]. In support of this idea the screening assays showed that the metabolites M1, and M2 were active against *An. stephensi* mosquitoes. In addition, M4 and M6 exhibited minor mosquitocidal properties either because the systemic exposure was lower or the metabolites are less mosquitocidal. M4 underwent demethylation on the disaccharide and hydroxylation on the hexahydrobenzofuran moiety like M1 and M2, respectively. All metabolites, which were hydroxylated on the spiroketal portion deglycosylated or both, were not or only slightly active (M6) at concentrations observed in clinical pharmacokinetic samples. However, the results presented here do not exclude that those metabolites are active at increased concentrations. In this study, a 3-day-LC_50_ of about 3 ng/mL was measured for ivermectin, which is slightly lower compared to observations made by Dreyer et al., with a 4-day-LC_50_ of 7 ng/mL against *An. stephensi* [12]. The LC_50_ value varies greatly between *Anopheles* species, insectary conditions, and with the feeding method or observation period. In *An. gambiae*, it was reported that the 7-day-LC_50_ of ivermectin is 3.4 ng/mL with blood fed from treated humans, and 15.9 ng/mL from spiked ivermectin experiments (in vitro mixture) [3]. In addition, *An. stephensi* were more susceptible to ivermectin in comparison to assays done with other *Anopheles* species [3]. Nonetheless, results were largely comparable to previous studies considering that the assay settings and conditions were not standardised (e.g. mosquito age, species, assay observation period). Further research is, however, needed to explore ivermectin metabolites impact on survival of other *Anopheles* species.

The current study estimated that M1 and M2 stay on average 69 h and 34 h above their LC_50_ value, and thus contribute significantly to the overall mosquito-lethal effect of ivermectin. This time above the lethality target can be considered as a “mosquitocidal window” of ivermectin, M1 and M2. The mosquitocidal window of ivermectin after a single oral 150 µg/kg dose for a vector with a LC_50_ of 6 ng/mL was predicted to be of 55 h and 7 h for a vector with a LC_50_ of 25 ng/mL [4]. Several pharmacological strategies can increase the duration of time above the LC_50_ and thus ivermectin’s efficacy in killing mosquitoes such as increasing the dose of ivermectin, employing repeated dosing regimens, and using a long-lasting drug formulation) [59]. Since M1 was also detected in livestock (e.g. pigs, goats, and sheep), the findings reported here might also be of relevance for livestock treatment for malaria vector control and other veterinary applications [50, 54]. Studies against nematode worms and other parasites are needed to assess the veterinary importance of ivermectin metabolites.

The present study has three evident limitations. First, the isolated fractions were analysed by multiple reaction monitoring, which was needed to reach the sensitivity to measure low-abundant metabolites. However, this detection mode is very selective and may not have recorded co-eluting constituents of the different fractions. Consequently, the overall purity of the isolated fractions could not be determined and it cannot be excluded that undetected metabolites or byproducts of the bioassay may have caused the observed mosquitocidal effects. However, the preparation of nine different blank fractions would have been very laborious, complicated considerably by the execution of the activity assays and would have substantially increased the required amount of consumables. Moreover, most of the fractions used for the treatments were not entirely pure and contained residues of the previous fraction (Additional file 1: Fig. S1 and Table S4). The screening assay showed that the fractions 1–6 were not or only slightly active (M6), so further purification of these fractions was not considered necessary. Fraction 8 contained mainly M2 but also residues of M4, whereas fraction 7 contained no M2. Consequently, the mosquitocidal effect of fraction 8 cannot be solely attributed to M2. However, M2 appears to be more active than M4, since the mosquito mortality of fraction 8 was much more pronounced than of fraction 7. Fraction 9 was reasonably pure and contained mainly M1, therefore, its activity can mostly be attributed to M1. Lastly, the metabolites were detected based on the parent mass and fragment and no elaborate structure identification was performed by e.g. nuclear magnetic resonance (NMR) or high-resolution mass spectrometry. Therefore, structure confirmation of at least the most active metabolites M1 and M2 by adequate analytical methods or synthesis of the reference substances is required to substantiate the findings of this present study.

Secondly, we did not investigate phase II metabolism of ivermectin, even though conjugation with glucuronic and sulphuric acids has been identified in sheep [52]. The pharmacological and toxicological role of phase II ivermectin metabolites remains unknown. In addition, Tipthara et al. identified further ivermectin metabolites, namely M10 and M12 detected in vitro after incubation with CYP3A4 microsomes and M13 with CYP2C8. However, these metabolites were not detectable in hepatocytes or blood. Hence, it may be argued whether to assess or not the mosquitocidal activity of these metabolites in further work.

Finally, the pharmacological activity of the metabolites was investigated using levels monitored in humans after the application of a therapeutic ivermectin dose. The tested metabolite concentration had to be matched with C_max_ levels obtained from pharmacokinetic profiles because no references were available. Hence, the applied metabolite quantity used to treat the mosquitoes is unknown. It cannot be ruled out that metabolites judged as inactive in the presented setup might be active at higher levels and possibly also more potent than ivermectin itself. In addition, the bioassay recorded the effect over three days, which is shorter compared to assays employed by others [11]. It is possible that more pronounced effects would have been detected for e.g. M4 and M6 by prolonging the duration of the assay. Still, the findings suggest that mainly M1 and M2 reach pharmacologically relevant concentrations in vivo and contribute to the mosquitocidal activity of ivermectin treatments.

Nonetheless, if metabolite activity or pharmacokinetic properties such as extended effect—not only against arthropods, but also in other indications—prove to be clinically relevant, future studies could explore the use of co-medications that interact with the metabolism of ivermectin. For instance, co-administering inhibitors of CYP3A may boost exposure to the parent drug, whereas inducers of the same pathway could increase production of active metabolites. Less toxic inhibitors of CYP3A and P-gp, for instance low dose ritonavir or cobicistat, should be considered.

## Conclusions

In conclusion, this study established a protocol to produce, isolate and test the activity of nine ivermectin metabolites. These results demonstrate that ivermectin metabolites desmethyl-H_2_B_1a_ (M1) and hydroxy-H_2_B_1a_ (M2) are active against *An. stephens*i at concentrations attained in the blood circulation of humans treated with a regular oral dose of 12 mg ivermectin. Pharmacologically relevant amounts of metabolites were used to allow the prediction of their in vivo activity, circumventing laborious and costly synthesis of reference substances. The methodology developed here can be applied to assess the activity of ivermectin metabolites against other parasites, which may further the development of novel chemotherapies. Overall, this study provides a better understanding of the pharmacokinetics of ivermectin metabolites and might facilitate further insight into efficacy for neglected tropical diseases and malaria, as ivermectin has been suggested as a possible vector control tool to assist malaria elimination. This approach can be applied to other drugs to study the activity of their metabolites. It should be emphasised that actual concentrations would still need to be assessed in PK trials to understand if relevant exposures can be achieved. For complex small molecular drugs such as ivermectin, the synthesis of references can generate considerable costs and operational complexity. The described approach adds value as a directed screening tool to focus resources on the most promising candidates.

## Supplementary Information

**Additional file 1: Table S1.** Specific tandem mass spectrometry settings applied for the detection and quantification of ivermectin and its metabolites. **Table S2.** Dual binary gradient flow program used for metabolism and fractioning assays. **Table S3.** Dual binary gradient flow program used for metabolism assays. **Figure S1.** Chromatogram before/after fractioning of the ivermectin metabolites. **Table S4.** Ivermectin metabolite compositionof each fraction. **Table S5.** Comparison of ivermectin metabolite C_max_ pharmacokinetic levels with spiked blank blood samples. **Figure S2.** Screening for ivermectin metabolites effect on mosquito activity. **Figure S3.** Fit of the nonlinear model to calculate ivermectin, M1 and M2 LC50. **Figure S4.** Compound level above LC_50_ in whole blood for ivermectin, desmethyl-H_2_B_1a_ (M1) and hydroxy-H_2_B_1a_ (M2).

## Data Availability

All data generated or analysed during this study are included in this published article and its supplementary information files.

## Abbreviations

CYP 3A4/5: Cytochrome P_450_ 3A4/5
C_max_: Maximal blood concentration
LC_50_: Lethal concentration that kills 50% of mosquitoes
LC–MS/MS: Liquid chromatography tandem mass spectrometry
M1: Desmethyl-H_2_B_1a_
M2: Hydroxy-H_2_B_1a_
M3: Hydroxy-H_2_B_1a_
M4: Desmethyl, hydroxy-H_2_B_1a_
M5: Hydroxy-H_2_B_1a_ monosaccharide
M6: Desmethyl, hydroxy-H_2_B_1a_
M7: Hydroxy-H_2_B_1a_ monosaccharide
M8: Dihydroxy- H_2_B_1a_
M9: Hydroxy-H_2_B_1a_
T_max_: Time of C_max_
