# Supplementary material for: Pharmacokinetics of ivermectin metabolites and their activity against Anopheles stephensi mosquitoes
Source: Malar J. 2023 Jun 24;22:194. doi: 10.1186/s12936-023-04624-0 (PMC10290335; doi:10.1186/s12936-023-04624-0)
Supplement: Supplementary file 1 — Additional file 1: Table S1. Specific tandem mass spectrometry settings applied for the detection and quantification of ivermectin and its metabolites. Table S2. Dual binary gradient flow program used for metabolism and fractioning assays. Table S3. Dual binary gradient flow program used for metabolism assays. Figure S1. Chromatogram before/after fractioning of the ivermectin metabolites. Table S4. Ivermectin metabolite compositionof each fraction. Table S5. Comparison of ivermectin metabolite Cmax pharmacokinetic levels with spiked blank blood samples. Figure S2. Screening for ivermectin metabolites effect on mosquito activity. Figure S3. Fit of the nonlinear model to calculate ivermectin, M1 and M2 LC50. Figure S4. Compound level above LC50 in whole blood for ivermectin, desmethyl-H2B1a (M1) and hydroxy-H2B1a (M2). [file 12936_2023_4624_MOESM1_ESM.pdf]

Supplements

List of supplementary figures and tables:

Table S1 Specific tandem mass spectrometry settings applied for the detection and quantification of ivermectin and its metabolites.
Table S2 Dual binary gradient flow program used for metabolism and fractioning assays.
Table S3 Dual binary gradient flow program used for metabolism assays.
Figure S1 Chromatogram before/after fractioning of the ivermectin metabolites.
Table S4 Ivermectin metabolite composition (M1-M9) of each fraction (F1-F9).
Table S5 Comparison of ivermectin metabolite C_{max} pharmacokinetic levels with spiked blank blood samples.
Figure S2 Screening for ivermectin metabolites effect on mosquito activity.
Figure S3 Fit of the nonlinear model to calculate IVM, M1 and M2 LC_{50}.
Figure S4 Compound level above LC_{50} in whole blood for ivermectin (IVM), desmethyl-H_2B_1a (M1) and hydroxy-H_2B_1a (M2).

Table S1 – Specific tandem mass spectrometry settings applied for the detection and quantification of ivermectin and its metabolites.

Analyte	Q1 Mass [Da]	Q3 Mass [Da]	Dwell Time [ms]	DP [V]	EP [V]	CE [V]	CXP [V]
**Ivermectin I**	892.4	569.2	10	106	10	17	30
**Ivermectin II**	892.4	307.1	10	106	10	31	20
**Ivermectin-d_2 I**	894.4	571.3	10	131	10	17	30
**Ivermectin-d_2 II**	894.4	309.2	10	131	10	29	26
**M1**: desmethyl-H_2B_{1a}	878.0	307.1	10	106	10	31	20
**M2**: hydroxy-H_2B_{1a}	908.0	307.1	10	106	10	31	20
**M3**: hydroxy-H_2B_{1a}	908.0	323.0	10	106	10	31	20
**M4**: desmethyl, hydroxy-H_2B_{1a}	894.0	307.0	10	106	10	31	20
**M5**: hydroxy-H_2B_{1a} monosaccharide	764.0	307.0	10	106	10	31	20
**M6**: desmethyl, hydroxy-H_2B_{1a}	894.0	323.0	10	106	10	31	20
**M7**: hydroxy-H_2B_{1a} monosaccharide	764.0	323.0	10	106	10	31	20
**M8**: dihydroxy-H_2B_{1a}	924.0	323.0	10	106	10	31	20
**M9**: hydroxy-H_2B_{1a}	908.1	323.0	10	106	10	31	20

H_2B_{1a}: ivermectin, mass filters (Q1 and Q3), Da: Daltons, ms: milliseconds, V: volts, DP: declustering potential, EP: entrance potential, CE: collision energy, CXP: Collision Cell Exit Potential.

Table S2 – Dual binary gradient flow program used for metabolism and fractioning assays.
Sample was loaded onto the analytical column using 2% mobile phase B. The initial total flow 1 and 3 was 0.1 mL/min (pump A and B) and 0.5 mL/min (pump C and D), respectively. Samples were diluted with mobile phase A within a T-union installed in front of the analytical column by pump C (total flow 3).

Time [min]	HPLC Module	Event	Parameter
**0.50**	Pumps	Pump B Conc.	2%
**0.50**	Pumps	Total Flow 1	0.6 mL/min
**0.50**	Pumps	Total Flow 3	0 mL/min
**1.50**	Pumps	Pump B Conc.	70%
**2.00**	Oven	Right Valve	0
**9.00**	Pumps	Pump B Conc.	87%
**9.01**	Pumps	Pump B Conc.	95%
**9.50**	Oven	Right Valve	1
**11.50**	Pumps	Pump B Conc.	95%
**11.51**	Pumps	Pump B Conc.	2%
**12.00**	Controller	Stop	

Pump B Conc.: Concentration of mobile phase B delivered by pump A and B.
Total Flow 1: Flow of pump A and B.
Total Flow 3: Flow of pump C and D.
For metabolism assays, the HPLC flow was directed into the MS (right valve: position 0) between 2.0 – 9.5 min, otherwise directed into the solvent waste (right valve: position 1).

Table S3 – Dual binary gradient flow program used for pharmacokinetic assays.
Sample was loaded onto the analytical column using 2% mobile phase B. The initial total flow 1 and 3 was 0.1 mL/min (pump A and B) and 0.5 mL/min (pump C and D), respectively. Samples were online diluted with 2% mobile phase B (98% mobile phase A) within a T-union installed in front of the analytical column.

Time [min]	HPLC Module	Event	Parameter
**0.50**	Pumps	Pump B Conc.	2 %
**0.50**	Pumps	Total Flow 1	0.6 mL/min
**0.50**	Pumps	Total Flow 3	0 mL/min
**1.00**	Pumps	Pump B Conc.	60 %
**2.50**	Oven	Right Valve	0
**5.00**	Pumps	Pump B Conc.	82 %
**5.25**	Pumps	Pump B Conc.	95 %
**5.50**	Oven	Right Valve	1
**6.00**	Pumps	Pump B Conc.	95 %
**6.01**	Pumps	Pump B Conc.	2 %
**6.50**	Controller	Stop	

Pump B Conc.: Concentration of mobile phase B delivered by pump A and B.
Total Flow 1: Flow of pump A and B.
Total Flow 3: Flow of pump C and D.

The HPLC flow was directed into the MS (right valve: position 0) between 2.5 – 5.5 min, otherwise directed into the solvent waste (right valve: position 1).

Figure S1 – Chromatogram before/after fractioning of the ivermectin metabolites.

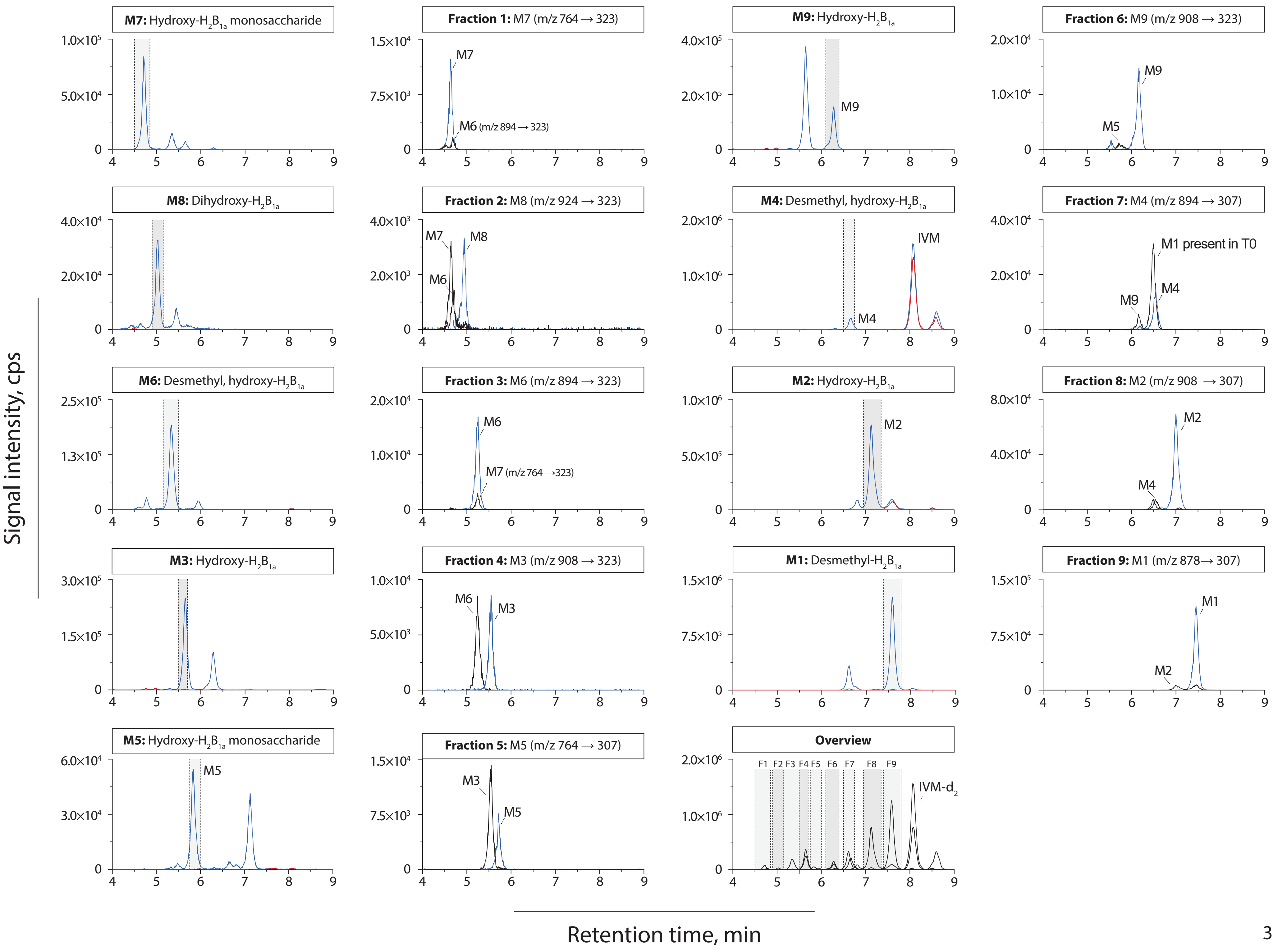

Table S4 – Ivermectin metabolite composition (M1-M9) of each fraction (F1-F9).
Numbers indicate the mean peak area (n=4) in percentage to the largest observed signal of the same metabolite. The standard deviation is given in parentheses. Values under 10% are not included. Bold numbers indicate which metabolite was considered for each fraction (e.g. 100% of M9 in fraction 6 [F6]).

	M7, %	M8, %	M6, %	M3, %	M5, %	M9, %	M4, %	M2, %	M1, %
**F1**: 4.50 – 4.85 min	**100**								
**F2**: 4.90 – 5.15 min	24.4 (5.4)	**100**							
**F3**: 5.15 – 5.50 min		21.1 (4.4)	**100**						
**F4**: 5.50 – 5.70 min			40.4 (6.5)	**66 (5.3)**					
**F5**: 5.75 – 6.00 min				100	**100**				
**F6**: 6.10 – 6.40 min					29.3 (5.7)	**100**			
**F7**: 6.50 – 6.75 min						28.9 (2.7)	**100**		
**F8**: 6.95 – 7.35 min					10.2 (2.2)		70.3 (9.9)	**100**	
**F9**: 7.40 – 7.80 min								13.3 (1.3)	**100**

Ivermectin (H_2B_{1a}) metabolites: **M1**: Desmethyl-H_2B_{1a}, **M2**: Hydroxy-H_2B_{1a}, **M3**: Hydroxy-H_2B_{1a}, **M4**: Desmethyl, hydroxy-H_2B_{1a}, **M5**: Hydroxy-H_2B_{1a} monosaccharide, **M6**: Desmethyl, hydroxy-H_2B_{1a}, **M7**: Hydroxy-H_2B_{1a} monosaccharide, **M8**: Dihydroxy-H_2B_{1a}, **M9**: Hydroxy-H_2B_{1a}.

Table S5a-c – Comparison of ivermectin metabolite C_{max} pharmacokinetic levels with spiked blank blood samples.
Maximal analyte peak area of metabolite 1-9 (M1-M9) measured for PK subject 1-12 in 50 µL blood. An aliquot of 50 µL blank blood of PK subject 2, 4, and 6 was spiked with 10 µL of metabolite fraction (F1: M7, F2: M8, F3: M6, F4: M3, F5: M5, F6: M9, F7: M4, F8: M2, F9: M1). The mean peak area of the spiked blood samples was compared with the mean area of the C_{max} samples to calculate the volume of metabolite fraction required to obtain equal values.

Table S5a – Data of ivermectin metabolites M1-M3

PK Subject ID	Observed C_{max} of M1	M1 spiked to blank blood	Observed C_{max} of M2	M2 spiked to blank blood	Observed C_{max} of M3	M3 spiked to blank blood
	Peak area (counts)	Peak area (counts)	Peak area (counts)	Peak area (counts)	Peak area (counts)	Peak area (counts)
1	1.95E+04		1.84E+04		6.21E+03	
2	1.01E+04	5.07E+04	9.36E+03	3.57E+04	3.33E+03	2.97E+03
3	1.39E+04		1.31E+04		4.98E+03	
4	8.48E+03	6.10E+04	7.42E+03	4.78E+04	2.70E+03	3.46E+03
5	9.52E+03		9.63E+03		3.63E+03	
6	2.33E+04	7.58E+04	2.00E+04	3.11E+04	5.52E+03	3.35E+03
7	1.56E+04		1.39E+04		4.44E+03	
8	1.79E+04		1.25E+04		4.04E+03	
9	1.28E+04		1.08E+04		3.41E+03	
10	1.60E+04		1.50E+04		4.42E+03	
11	2.28E+04		1.64E+04		4.22E+03	
12	1.78E+04		1.59E+04		4.51E+03	
Mean	1.56E+04	6.25E+04	1.35E+04	3.82E+04	4.28E+03	3.26E+03
Difference spiked blood / C_{max}		4.0		2.8		0.8
Vol. of fraction required for 50 µL blood		2.5		3.5		13.1
Vol. of fraction required for 3 mL blood		**150**		**212**		**788**

Ivermectin (H_2B_{1a}) metabolites: **M1**: Desmethyl-H_2B_{1a}, **M2**: Hydroxy-H_2B_{1a}, **M3**: Hydroxy-H_2B_{1a}, **M4**: Desmethyl, hydroxy-H_2B_{1a}, **M5**: Hydroxy-H_2B_{1a} monosaccharide, **M6**: Desmethyl, hydroxy-H_2B_{1a}, **M7**: Hydroxy-H_2B_{1a} monosaccharide, **M8**: Dihydroxy-H_2B_{1a}, **M9**: Hydroxy-H_2B_{1a}.

Table S5b – Data of ivermectin metabolites M4-M6.

PK Subject ID	Observed C_{max} of M4	M4 spiked to blank blood	Observed C_{max} of M5	M5 spiked to blank blood	Observed C_{max} of M6	M6 spiked to blank blood
	Peak area (counts)	Peak area (counts)	Peak area (counts)	Peak area (counts)	Peak area (counts)	Peak area (counts)
1	6.13E+03		7.86E+02		3.04E+03	
2	4.65E+03	8.84E+03	5.85E+02	2.73E+03	1.56E+03	1.03E+04
3	5.03E+03		1.01E+03		2.10E+03	
4	4.08E+03	1.01E+04	7.22E+02	3.40E+03	1.60E+03	6.99E+03
5	2.43E+03		6.06E+02		1.15E+03	
6	7.98E+03	5.39E+03	1.25E+03	3.27E+03	3.23E+03	7.74E+03
7	4.09E+03		8.58E+02		1.62E+03	
8	5.21E+03		6.84E+02		1.82E+03	
9	4.12E+03		6.59E+02		1.80E+03	
10	5.38E+03		7.23E+02		1.99E+03	
11	5.67E+03		8.95E+02		2.12E+03	
12	5.92E+03		7.29E+02		2.69E+03	
Mean	5.06E+03	8.11E+03	7.92E+02	3.13E+03	2.06E+03	8.33E+03
Difference spiked blood / C_{max}		1.6		4.0		4.0
Vol. of fraction required for 50 µL blood		6.2		2.5		2.5
Vol. of fraction required for 3 mL blood		**374***		**152**		**148**

Ivermectin (H_2B_{1a}) metabolites: **M4**: Desmethyl, hydroxy-H_2B_{1a}, **M5**: Hydroxy-H_2B_{1a} monosaccharide, **M6**: Desmethyl, hydroxy-H_2B_{1a},

Table S5c – Data of ivermectin metabolites M7-M9.

**PK Subject ID**	**Observed C_{max} of M7** Peak area (counts)	**M7 spiked to blank blood** Peak area (counts)	**Observed C_{max} of M8** Peak area (counts)	**M8 spiked to blank blood** Peak area (counts)	**Observed C_{max} of M9** Peak area (counts)	**M9 spiked to blank blood** Peak area (counts)
1	1.31E+03		7.02E+02		N/A	
2	7.70E+02	5.49E+03	5.34E+02	2.05E+03	N/A	1.09E+04
3	9.70E+02		4.91E+02		N/A	
4	7.68E+02	6.70E+03	3.41E+02	1.37E+03	N/A	1.24E+04
5	8.12E+02		3.43E+02		N/A	
6	1.39E+03	4.86E+03	6.41E+02	1.54E+03	N/A	1.08E+04
7	1.26E+03		3.42E+02		N/A	
8	1.09E+03		4.91E+02		N/A	
9	7.88E+02		4.29E+02		N/A	
10	9.63E+02		4.93E+02		N/A	
11	1.21E+03		2.79E+02		N/A	
12	9.57E+02		4.55E+02		N/A	
Mean	1.02E+03	5.68E+03	4.62E+02	1.65E+03	N/A	1.14E+04
Difference spiked blood / C_{max}		5.5		3.6		N/A
Vol. of fraction required for 50 µL blood		1.8		2.8		N/A
Vol. of fraction required for 3 mL blood		**108**		**168**		**120***

Ivermectin (H_2B_{1a}) metabolites: **M7**: Hydroxy-H_2B_{1a} monosaccharide, **M8**: Dihydroxy-H_2B_{1a}, **M9**: Hydroxy-H_2B_{1a}.

Figure S2 – Screening for ivermectin metabolites effect on mosquito activity.

Anopheles stephensi mosquitoes were treated with blank human blood (**blank**), or blood containing either ivermectin (**IVM**, 50 ng/mL), or an ivermectin metabolite (M1-M9): **M1**: Desmethyl-H_2B_{1a}, **M2**: Hydroxy-H_2B_{1a}, **M3**: Hydroxy-H_2B_{1a}, **M4**: Desmethyl, hydroxy-H_2B_{1a}, **M5**: Hydroxy-H_2B_{1a} monosaccharide, **M6**: Desmethyl, hydroxy-H_2B_{1a}, **M7**: Hydroxy-H_2B_{1a} monosaccharide, **M8**: Dihydroxy-H_2B_{1a}, **M9**: Hydroxy-H_2B_{1a}.

Three batches of mosquitoes, with an average of 26 mosquitoes/condition, were used per treatment group. Mean mosquito activity was assessed after 24h, 48h and 72h. Error bars correspond to the range. Mosquito's activity score: +2: The mosquito can fly and reach the top of the cup, it stays mainly on the top of the cup; +1: The mosquito is staying on the bottom of the cup, it is not able to fly but maybe can cover short distances on the ground; 0: No movements observed upon physical contact, the mosquito is classified as dead.

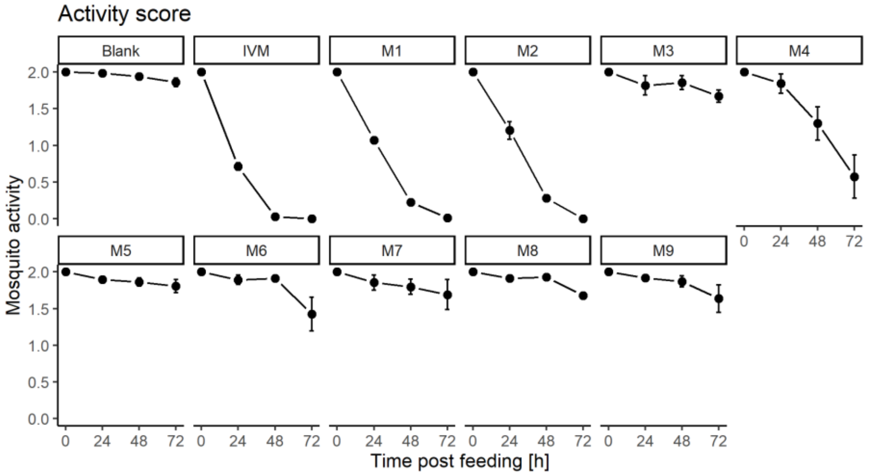

IVM metabolite	Time post feeding [h]	Average activity score	SE	IVM metabolite	Time post feeding [h]	Average activity score	SE
**IVM**	0	2.00	0.00	**M5**	0	2.00	0.00
**IVM**	24	0.71	0.05	**M5**	24	1.90	0.01
**IVM**	48	0.03	0.02	**M5**	48	1.86	0.06
**IVM**	72	0	0.00	**M5**	72	1.81	0.09
**M1**	0	2.00	0.00	**M6**	0	2.00	0.00
**M1**	24	1.07	0.04	**M6**	24	1.89	0.07
**M1**	48	0.23	0.02	**M6**	48	1.91	0.01
**M1**	72	0.01	0.01	**M6**	72	1.42	0.23
**M2**	0	2.00	0.00	**M7**	0	2.00	0.00
**M2**	24	1.20	0.12	**M7**	24	1.85	0.11
**M2**	48	0.28	0.05	**M7**	48	1.80	0.10
**M2**	72	0	0.00	**M7**	72	1.69	0.21
**M3**	0	2.00	0.00	**M8**	0	2.00	0.00
**M3**	24	1.82	0.13	**M8**	24	1.91	0.01
**M3**	48	1.86	0.09	**M8**	48	1.93	0.02
**M3**	72	1.67	0.08	**M8**	72	1.68	0.04
**M4**	0	2.00	0.00	**M9**	0	2.00	0.00
**M4**	24	1.84	0.13	**M9**	24	1.92	0.03
**M4**	48	1.30	0.23	**M9**	48	1.87	0.08
**M4**	72	0.57	0.29	**M9**	72	1.64	0.19
**blank**	0	2.00	0.00				
**blank**	24	1.98	0.02				
**blank**	48	1.94	0.04				
**blank**	72	1.86	0.06				

Figure S3 – Fit of the non-linear model to calculate IVM, M1 and M2 LC_{50}.
Multiple doses/concentrations of drug (**IVM:** ivermectin, **M1**: Desmethyl-H_2B_{1a}, **M2**: Hydroxy-H_2B_{1a}) were orally fed to *Anopheles stephensi* mosquitoes to determine the lethal concentration that killed 50% of the mosquitoes (LC_{50}) 72h after treatment. The blood samples of the participants of the pharmacokinetics trial (Duthaler et al., 2019) were analyzed to derive the maximal blood level (C_{max}) for ivermectin, M1 and M2 **(Figure 2)**. An administration of 12 mg ivermectin yielded a C_{max} of 50 ng/mL for ivermectin, therefore LC_{50} was evaluated using ivermectin concentrations ranging between 1–12.5 ng/mL. For the metabolites M1 and M2, C_{max} corresponds to the maximal intensity (peak area, counts) from the metabolite peak intensity time plots. *Anopheles stephensi* were treated with different dilutions of ivermectin ($^1/_4$ C_{max}, $^1/_6$ C_{max}, $^1/_{10}$ C_{max}, $^1/_{20}$ C_{max}, $^1/_{50}$ C_{max}), M1 (C_{max}, $^1/_2$ C_{max}, $^1/_4$ C_{max}, $^1/_6$ C_{max}, $^1/_{10}$ C_{max}) and M2 (C_{max}, $^1/_2$ C_{max}, $^1/_3$ C_{max}, $^1/_4$ C_{max}, $^1/_5$ C_{max}). The experiment was performed in triplicate, with an average of 24 [8 – 54] mosquitoes per drug dilution and a total number of mosquito fed per drug (n). A plot of the observed mortality versus the dose/concentration fed to the mosquito (black dots) was overlaid with the plot of the confidence bands (grey) around the fitted regression curve (black line). LC_{50} summarized in **Table 2**.

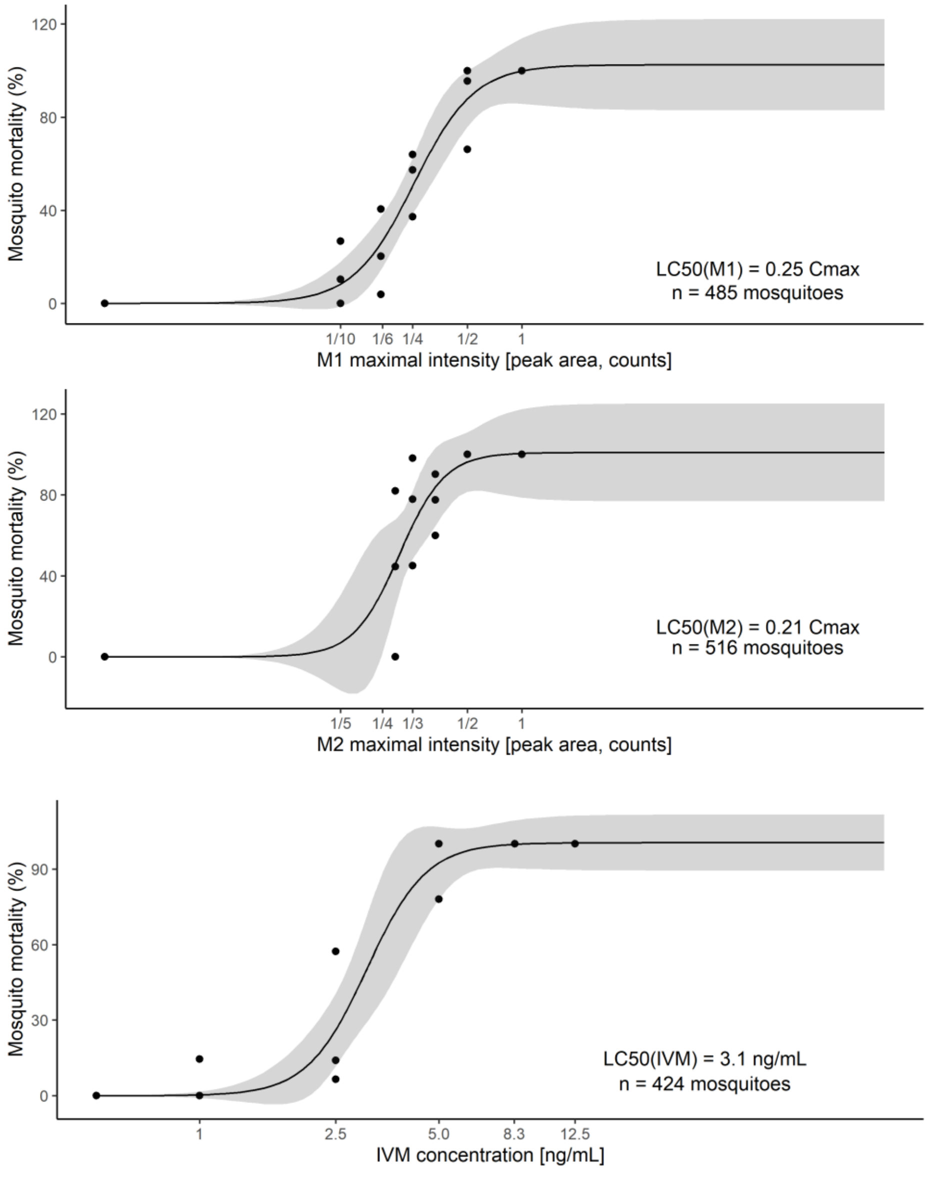

Figure S4 – Compound level above LC_{50} in whole blood for ivermectin (IVM), and metabolites desmethyl-H_2B_{1a} (M1) and hydroxy-H_2B_{1a} (M2).
The blood samples of the participants of the pharmacokinetics trial (Duthaler et al., 2019), pre- and up to 72h post-ivermectin dose, were analyzed to derive the pharmacokinetic profiles for ivermectin, M1 and M2. The LC_{50} values were computed in **Figure S3** and used to derive the time above LC_{50}.

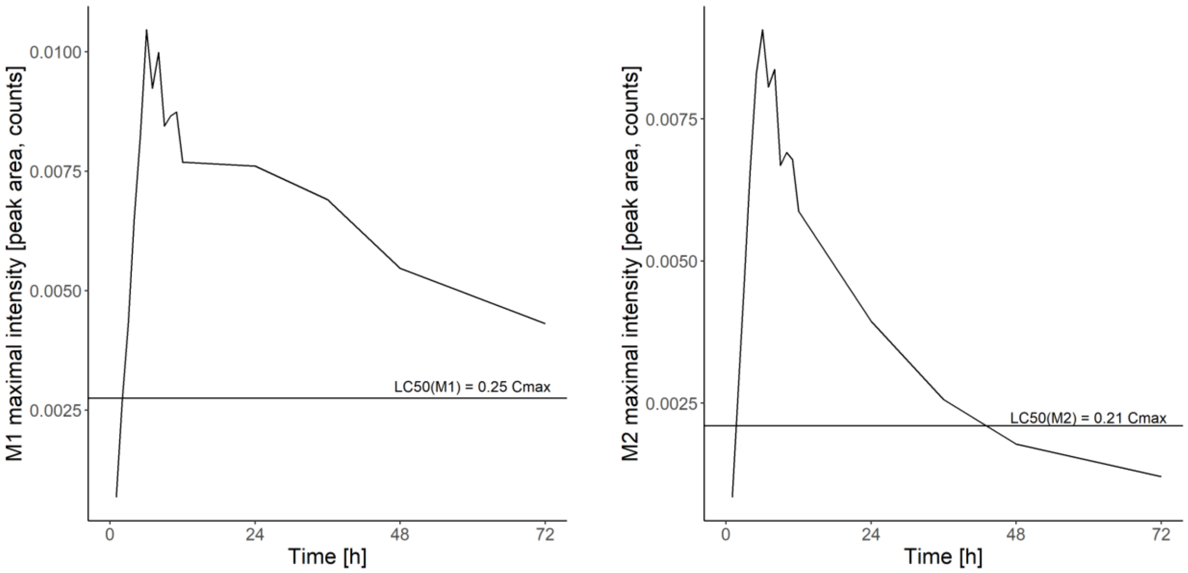

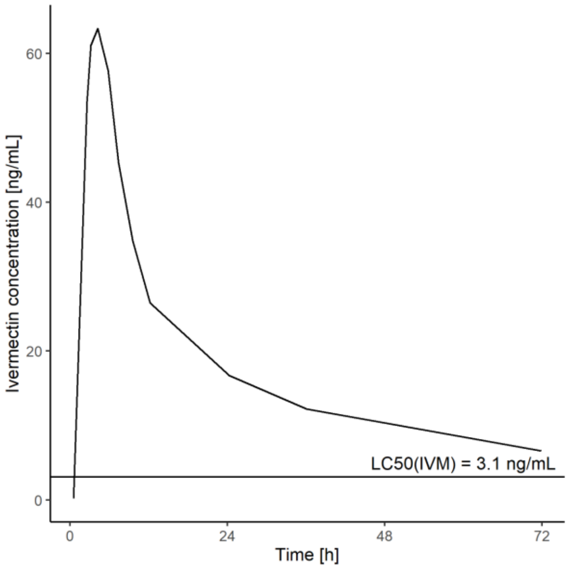

	Time above LC_{50} [h]
M1	69.0
M2	34.0
IVM	69.3
